# An Investigation of the Immune Microenvironment and Genome during Lung Adenocarcinoma Development

**DOI:** 10.7150/jca.92101

**Published:** 2024-01-27

**Authors:** Qingyi Wang, Bin Xie, Jingyue Sun, Zisheng Li, Desheng Xiao, Yongguang Tao, Xiaoling She

**Affiliations:** 1 Department of Pathology, School of Basic Medicine, Central South University, Changsha, Hunan, 410078, China.; 2 Department of Pathology, Xiangya Hospital, Central South University, Changsha, Hunan 410078 China.; 3 Cancer Research Institute, School of Basic Medicine, Central South University, Changsha, Hunan, 410078, China.; 4 Key Laboratory of Carcinogenesis and Cancer Invasion (Central South University), Ministry of Education, Hunan, 410078, China.; 5 Department of Pathology, The Second Xiangya Hospital, Central South University Changsha, Hunan, 410011, China.

**Keywords:** pre-invasive adenocarcinoma, immunoediting, tumor-infiltrating lymphocytes, tumor immune typing

## Abstract

**Background:** Adenocarcinoma in situ (AIS) and minimally invasive adenocarcinoma (MIA) are two consecutive pathological processes that occur before invasive lung adenocarcinoma (LUAD). However, our understanding of the immune editing patterns during the progression of LUAD remains limited. Furthermore, we know very little about whether alterations in driver genes are involved in forming the tumor microenvironment (TME). Therefore, it is necessary to elucidate the regulatory role of TME in LUAD development from multiple dimensions, including immune cell infiltration, molecular mutation events, and oncogenic signaling pathways.

**Methods:** We collected 145 surgically resected pulmonary nodule specimens, including 28 cases of AIS, 52 cases of MIA, and 65 cases of LUAD. Immunohistochemistry (IHC) was used to detect the expression of immune markers CD3, CD4, CD8, CD68 and programmed death ligand 1 (PD-L1). Genomic data and TMB generated by targeted next-generation sequencing (NGS).

**Results:** LUAD exhibited higher levels of immune cell infiltration, tumor mutation burden (TMB), and activation of oncogenic pathways compared to AIS and MIA. In LUAD, compared to epidermal growth factor receptor (EGFR) single mutation and wild-type (WT) samples, cases with EGFR co-mutations showed a more pronounced rise in the CD4/CD8 ratio and CD68 infiltration. Patients with low-density lipoprotein (LDL) receptor-related protein 1B (LRP1B) mutation have higher TMB and PD-L1 expression. The transition from AIS to LUAD tends to shift the TME towards the PD-L1^+^CD8^+^ subtype (adaptive resistance). Progression-associated mutations (PAMs) were enriched in the lymphocyte differentiation pathway and related to exhausted cells' phenotype.

**Conclusion:** Tumor-infiltrating immune cells tend to accumulate as the depth of LUAD invasion increases, but subsequently develop into an immune exhaustion and immune escape state. Mutations in EGFR and LRP1B could potentially establish an immune niche that fosters tumor growth. PAMs in LUAD may accelerate disease progression by promoting T cell differentiation into an exhausted state.

## Introduction

As the most common subtype of lung cancer, lung adenocarcinoma (LUAD) has many treatment options^1^. However, its prognosis remains poor, with a 5% survival rate over five years^2^. Due to the increased popularity of early diagnosis and lung cancer screening, more LUAD cases are detected at early stages, and surgical resection is employed to cure the disease^3^. Adenocarcinoma in situ (AIS) and minimally invasive adenocarcinoma (MIA) represent two consecutive stages that progress to LUAD^4^. From a radiological perspective, both AIS and MIA nodules are smaller than or equal to 3 cm. Still, the difference is that AIS does not invade the basement membrane and is classified as a pre-invasive lesion, whereas MIA shows an invasion of less than 5 mm^5^, ^6^. In the pre-invasive stages, the 100% 5-year overall survival rate of MIA and AIS was notably more excellent than that of LUAD (15%)^6^-^8^. Past genomic analyses of pre-invasive LUAD have revealed the characteristics of point mutations, copy number variations, and genomic evolutionary patterns during the progression of lung cancer^9^-^11^. On the other hand, The development of LUAD is intricately linked to alterations in the immune milieu, wherein the presence of immune cells infiltrating the tumor assumes significant importance^12^, ^13^. Tumor microenvironment (TME) exhibits a diverse and dynamic tumor ecosystem, suggesting that tumor heterogeneity and the growth of tumors are not exclusively driven by genetic changes. It also has the potential to function as an innovative therapeutic target 14-18. Therefore, a further understanding of the TME characteristics in the pre-invasive stage of LUAD can help us comprehend the occurrence and development of this disease.

Additionally, a strong correlation has been noted between tumor mutation burden (TMB) and immune responses or regulatory mechanisms linked with tumor-associated antigens. The relationship between TMB and several factors, such as the response to immune checkpoint blockade, specific driver mutations, and the extent of immune infiltration, has been shown in previous studies^18^-^21^. Nevertheless, there is still a lack of comprehensive knowledge regarding the longitudinal progression and interplay of these factors during the pre-invasive and early invasive stages of LUAD development.

This work involved the investigation of immunological milieu and genomics in a total of 145 samples of LUAD, MIA, and AIS. We attempted to 1) validate the characteristics of immune cell infiltration, TMB, and genomic changes in the process of LUAD formation; 2) detect the critical role of specific molecular mutation events in the tumor formation process accompanied by immune reprogramming; 3) classify all samples based on the expression status of CD8 score and programmed death ligand 1(PD-L1) expression to characterize different developmental stages and degree of malignancy in LUAD; 4) integrate our data with public databases for immune genomic analysis and identify gene sets that may have an impact on patient prognosis.

## Methods

### Patients and specimen collection

We consecutively collected 145 lung nodule samples from patients who underwent surgery at Xiangya Hospital from June 2021 to March 2022. Among these samples, we identified 28 cases of AIS, 52 cases of MIA, and 65 cases of LUAD, all of which were treated for the first time. Our exclusion criteria included mucinous LUAD, receipt of induction therapy, residual disease, pathologic stage III and IV disease. The original data of the clinical characteristics and genomic information of the samples are displayed in the supplemental materials. We have also obtained transcriptomic data of matched adjacent normal tissues from the GEO database for AIS, MIA and LUAD (GSE169033, GSE32863). To supplement the lack of survival data in our dataset, we referenced clinical information from the TCGA Pan-Cancer Atlas^22^ and GEO database (GSE136961). All the transcriptional raw data from the GEO database are displayed in the supplemental materials.

### Genomic analysis

We performed hybridization capture-based NGS using Illumina NovaSeq6000/NextSeq CN500 to identify 448 genes associated with diagnosis, treatment, and prognosis of tumors (developed by Aide Xiamen). TMB level is calculated as the total number of non-synonymous or all somatic mutations per one million bases (Mb) in the genome of each cancer patient. The TMB of LUAD patients receiving immune checkpoint inhibitors (ICIs) treatment is sourced from a public database^23^**,** CAMOIP is employed as a tool for data analysis and visualization^24^.

Progression-associated mutations (PAMs) are defined as the intersection of mutated genes in LUAD and MIA/AIS. We utilized GSCA to analyze the correlation between PAM and exhausted T cell infiltration, and identified 6 genes (CARD11, DNMT3A, MUC16, RBM10, TRRAP, ZFHX4) with the most significant correlation, which we designated as PAM-exausted. We performed gene enrichment analysis and immunogenomic analysis on PAMs using Metascape^25^ and Gene Set Cancer Analysis (GSCA)^26^. Additionally, the databases of Cbiopotal and GSCA were utilized for survival analysis. We selected seven classic cancer pathways, phosphatidylinositol 3-kinase (PI3K) -Akt, Ras, Cell cycle, TGF-beta, Notch, Wnt, and p53, which have been previously reported^27^. The relevant gene mutations involved in each pathway were used to calculate the number of pathways altered (NPA) for each patient.

### Immune histochemistry Analysis

Levels of PD-L1 expression (developed by Aide Xiamen), CD3, CD4, CD8, and CD68 were independently scored by two pathologists. For each sample, PD-L1 expression was determined by the tumor proportion score (TPS), which measures the percentage of tumor cells with complete or partial membrane staining relative to all the tumor cells, including negative and positive cells. The scores of CD3, CD4, CD8, and CD68 were assessed as the percentage of positive cells among nucleated cells in the stromal compartment of each specimen. The scoring was based on the proportion of positive cells, with less than 20%, 20%-40%, 40-60%, 60%-80%, and more than 80% of the total cells recorded as 0, 3, 6, 9, 12, respectively.

### Transcriptome analysis

The gene expression data for AIS, MIA and LUAD was obtained from the GEO database (GSE169033, GSE32863), and immune infiltration analysis was conducted using the CIBERSORT algorithm. Analysis results were performed in Hiplot Pro (https://hiplot.com.cn/), a comprehensive web service for biomedical data analysis and visualization. The analysis of programmed death-1 (PD-1), PD-L1 expression and immune cell infiltration in patients with low-density lipoprotein (LDL) receptor-related protein 1B (LRP1B) mutations is derived from the TCGA database^28^, and CAMOIP is used as a tool to analyze expression data.

### Survival analysis

We obtained progression-free survival (PFS) information for patients with LRP1B mutations who received ICIs treatment from a public database^23^, and conducted analysis and visualization using CAMOIP. For determining the efficacy of ICIs in patients, we considered PFS above the median as responsive and below the median as non-responsive. Additionally, we performed PFS and disease-free survival (DFS) analysis using cBioPortal for patients with PAMs in the TCGA Pan-Cancer database for NSCLC^22^.

### Statistical analysis

Statistical analysis was conducted by using GraphPad Prism software (version 7.00, GraphPad Software, La Jolla, CA), SPSS software (version 24.0, IBM Corp, Armonk, NY), and Hiplot pro. Column plots, scatter dots, and box plots were generated to indicate median values and 95% confidence intervals (CIs). The chi-square, Fisher exact, and Kruskal-Wallis tests were set to calculate the significance of the differences between different subsets. All reported p-values were two-tailed, and significant differences were defined as those with a p-value less than 0.05. The notations ns, *, **,***,**** represent p > 0.05, p ≤ 0.05, p ≤ 0.01, p ≤ 0.001, p ≤ 0.0001 respectively.

## Results

### Gradually enhanced immune response from pre-invasive to early invasive stage in LUAD

**Figure 1:**
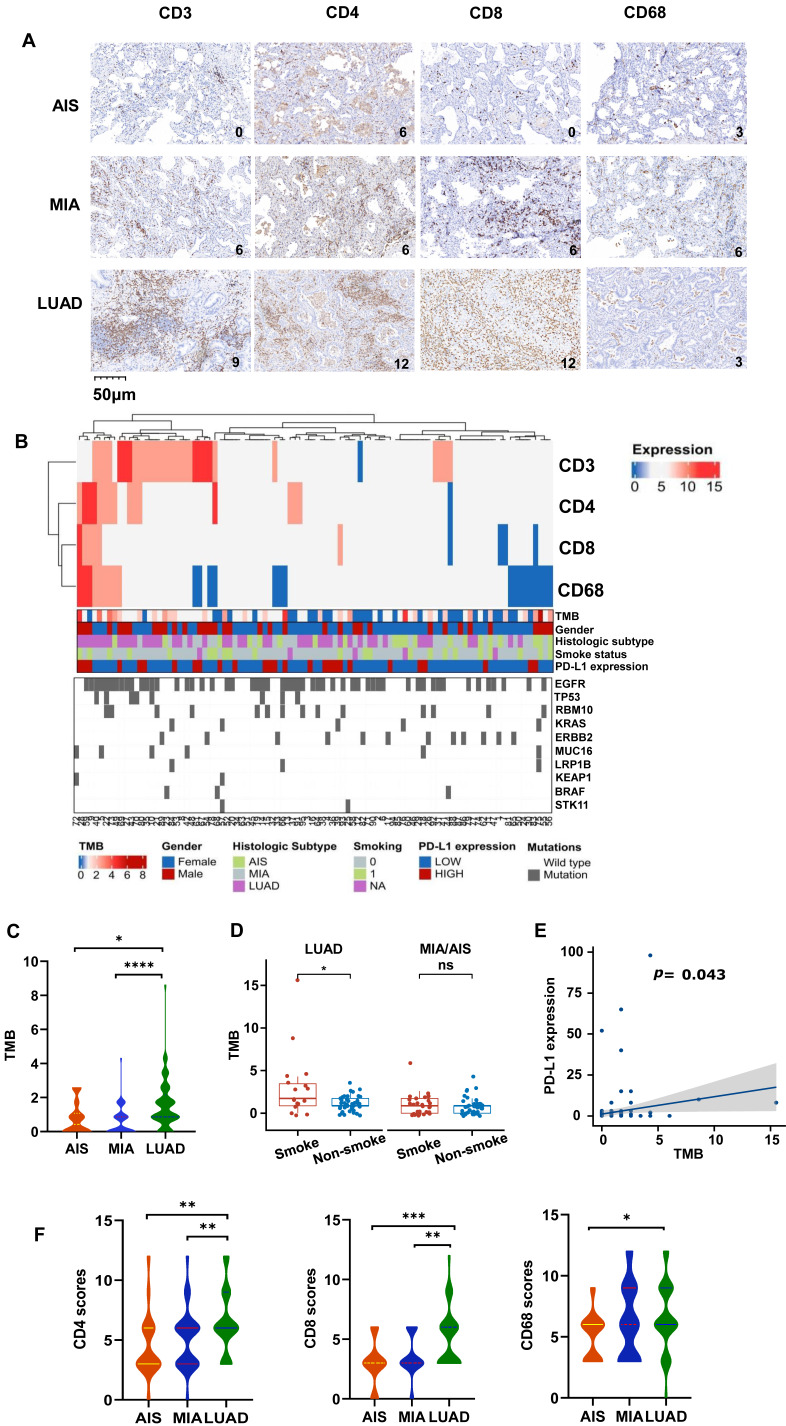
** Characteristic changes in the TME across different pathological types.** A. The representative immunohistochemical images of all subtypes. B. Comprehensive visualized plot based on LUAD and MIA/AIS patients. C. Comparison of TMB in AIS, MIA, and LUAD. D. Evaluation of TMB in smokers and non-smokers. E. Correlation analysis of TMB and PD-L1 expression in all subtypes. F. The scores of CD3+, CD4+, CD8+, and CD68+ cells infiltration in AIS, MIA, and LUAD.

To understand the overall characteristics during different stages of LUAD development, we first evaluated the immune cell markers of AIS, MIA, and LUAD in 145 cases. The scores of CD3, CD4, CD8, and CD68 was assessed to know the degree of tumor infiltration lymphocytes (TILs) or tumor-associated macrophages (TAMs) infiltration, which was graded 0-12 (Fig. 1A). As an immunosensitive indicator, the expression of PD-L1 was evaluated as positive (tumor proportion score (TPS)≥1%) and negative (TPS<1%) level. Furthermore, we integrated and summarized the available information by combining patient gender, smoking status, age, TMB level, histological type, and the top 10 genes ranked by mutation frequency and visualized it in a waterfall plot (Fig. 1B, Table 1). The distribution of males and females in LUAD was similar, accounting for 52.3% and 47.7%, respectively. Despite the lack of statistical significance, a higher proportion of females was observed in MIA (69.2%) and AIS (67.9%) compared to males (30.8% and 32.1%, respectively), potentially attributed to the elevated detection rate of pulmonary nodules in Asian women^29^. For detailed clinicopathological information, refer to Table 1.

The levels of TMB were compared among all subtypes, which is a crucial factor influencing outcomes and prognoses for non-small cell lung cancer (NSCLC) patients. LUAD had significantly higher TMB compared to AIS and MIA (Fig. 1C). In LUAD, smokers exhibited higher TMB levels than non-smokers (Fig. 1D), and all subtypes showed a positive correlation between PD-L1 expression and TMB level (Fig. 1E). However, there was no association between TMB and the expression of CD3, CD4, CD8, and CD68 (Table S1). Additionally, the CD3, CD4, CD8, and CD68 scores are higher in LUAD (Fig. 1F, Fig. S1A), along with higher TMB. We obtained the RNA expression data of AIS, MIA, and LUAD tumor samples from the TCGA database, as well as their matched adjacent normal samples (GSE169033, GSE32863). We conducted immune cell infiltration analysis and found that, in line with our results, as the invasiveness increased, the degree of immune cell infiltration deepened (Fig. S1B, S1C). It is noteworthy that we observed a significant increase in the infiltration of CD4 and CD8 lymphocytes in the adjacent normal tissue (Fig. S1C), which may suggest that immune cells may have infiltrated around the lesions in the early stages of tumor development. Consistently, Further analysis displayed low CD3 scores (0,3) accounted for a higher proportion in MIA/AIS. In contrast, high CD3 scores (6,9,12) accounted for an increased ratio in LUAD (Fig. S1D). We could still observe in the figure that the proportion of high PD-L1 expression in LUAD was relatively elevated (Fig. S1E). This proves that with the deepening of tumor invasion, immune infiltration is also enhanced.

### Oncogenic pathway analysis in LUAD and MIA/AIS

**Figure 2:**
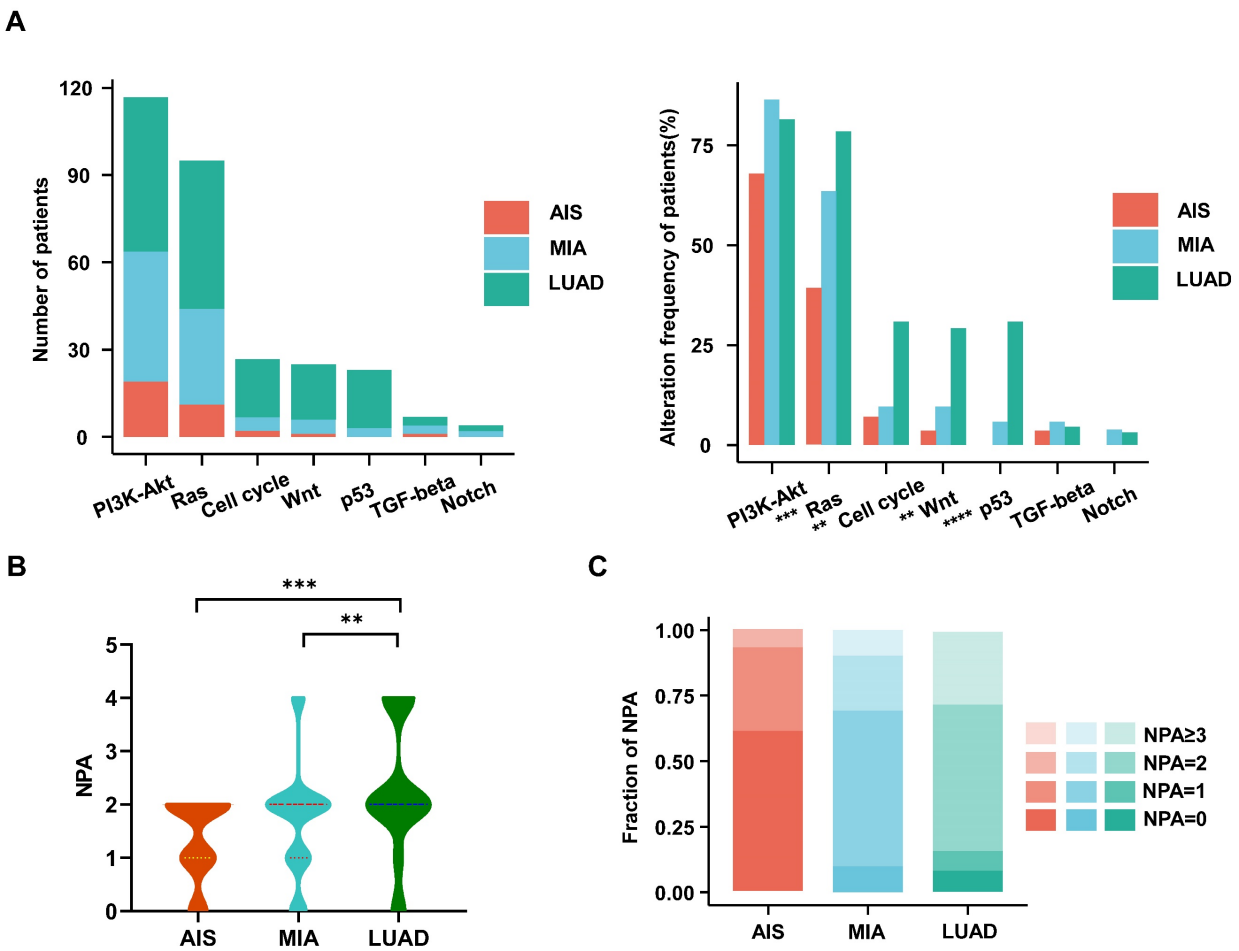
** Characteristics of the oncogenic pathways in the evolution of LUAD.** A. The bar chart shows the number (left) and the proportion (right) of patients with different alterations in oncogenic pathways in AIS, MIA, and LUAD. B. Violin plot shows the distribution of the number of pathway alterations (NPA) among different subtypes. C. The fraction of NPA according to histologic subtypes.

We investigated the frequencies of alterations in seven oncogenic signaling pathways, which have been previously reported^27^. The phosphatidylinositol 3-kinase (PI3K)-Akt pathway, an essential pathway regulating cell growth and metabolism, has been reported to be common in NSCLC patients^30^. It exhibited the highest frequency of alterations among all oncogenic pathways in our cohort. The Ras (79% vs. 64% vs. 39%; p<0.001), cell cycle (31% vs. 10% vs. 7%; p<0.003), Wnt (29% vs. 10% vs. 4%; p<0.002), and p53 (31% vs. 6% vs. 0%; p<0.0001) pathways had significantly higher alteration frequencies in LUAD than in MIA or AIS (Fig. 2A). Moreover, we observed that the number of pathways altered (NPA) of LUAD is significantly higher than that of MIA (p=0.0066) and AIS (p=0.00063) (Fig. 2B). AIS samples had a single NPA most frequently (n=8 [30%]). In comparison, tumors with ≥3 NPA occurred most often in LUAD (n=18 [27%])(Fig. 2C) (Table S2). Our data suggests that the proportion of pathway activation increases from AIS to LUAD subtypes.

### Somatic mutational signature in LUAD and MIA/AIS

**Figure 3:**
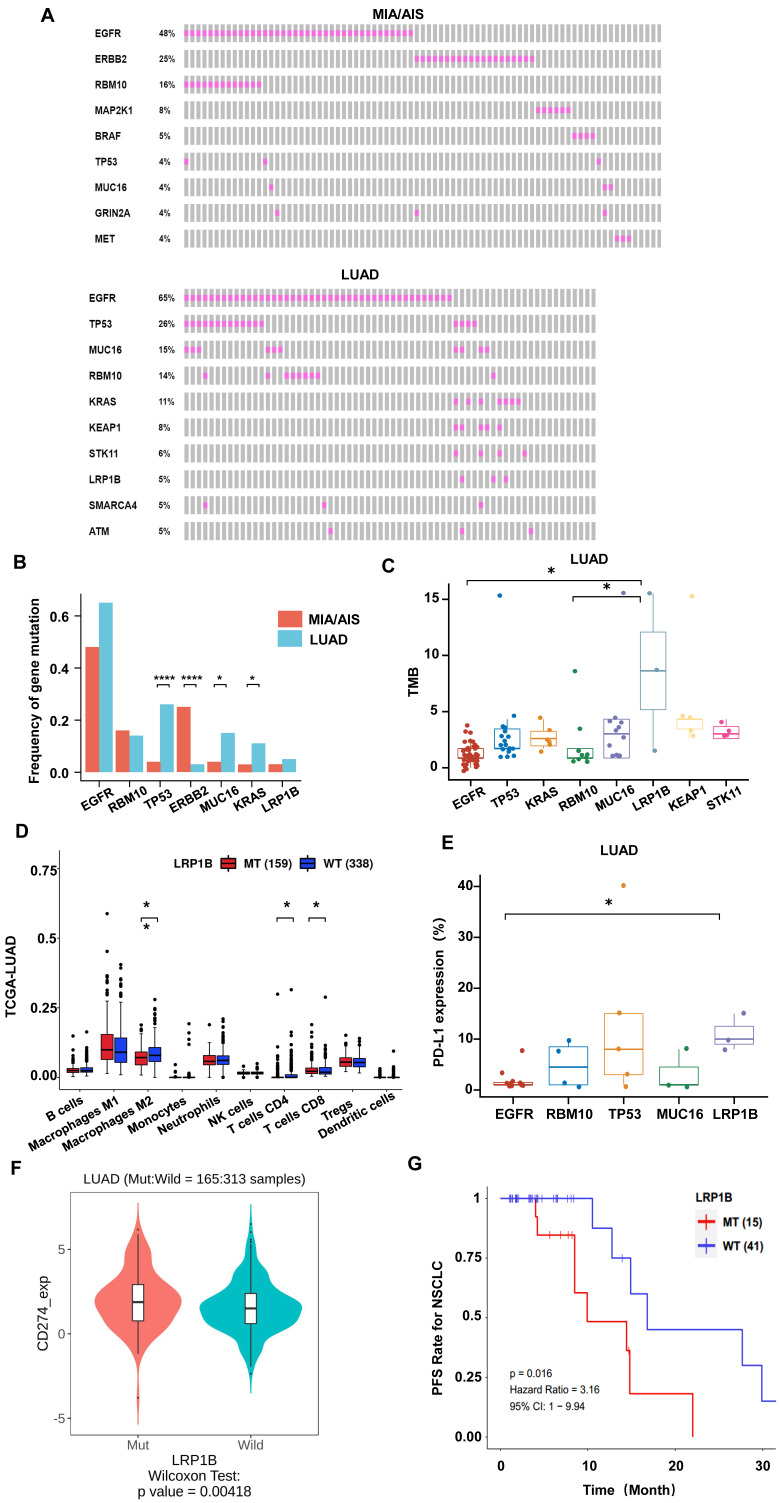
** Gene mutational landscape among pre-invasive and invasive LUAD.** A, B. Gene mutation spectrum of the top 10 mutated genes(A) and mutation frequency of high-frequency mutated genes(B) in MIA/AIS and LUAD, respectively. C. TMB levels of samples with high-frequency mutations in LUAD. D. Immune cell infiltration in patients with LRP1B mutation in LUAD E. The expression of PD-L1 of high-frequency mutated genes in LUAD (only PD-L1 positive). F. The PD-L1 expression in LRP1B-mutated and wild-type LUAD patients. G. The progression-free survival (PFS) of LRP1B-mutated and wild-type LUAD patients receiving immune checkpoint inhibitors (ICIs).

We performed whole-exome sequencing (WES) of 448 cancer-related genes on all samples. Epidermal growth factor receptor (EGFR) was identified as the most frequently mutated gene in both LUAD and MIA/AIS (Fig. 3A), with a frequency of 65% versus 48% (P=0.33). Apart from EGFR, TP53 (26%), MUC16 (15%), RNA-binding motif protein 10 (RBM10) (14%), and KRAS (11%) were the most frequently mutated genes in LUAD, while ERBB2 (25%), RBM10 (16%), MAP2K1 (8%), and BRAF (5%) were the most frequently mutated genes in MIA/AIS. Furthermore, we found that the mutation frequency of TP53 (26% vs. 4%, P<0.0001), MUC16 (15% vs. 4%, P=0.016), and KRAS (14% vs. 3%, P=0.043) was higher in LUAD than in MIA/AIS. And a higher incidence of ERBB2 mutations in MIA/AIS compared to LUAD (25% vs 3%, P<0.0001) (Fig. 3B). This preliminary reflects the unique molecular characteristics within different pathological stages of LUAD.

We then investigated the association between high-frequency mutations and TMB. Notably, LRP1B mutated samples displayed a substantially higher TMB (Fig. 3C, S2A). We further analyzed the immune cell infiltration in LUAD patients with LRP1B mutation and wild-type (WT) in the TCGA database^28^, and found that patients with mutation have lower infiltration of macrophages and CD4+ T cells, and higher infiltration of CD8 T cells (Fig. 3D). On the other hand, in the LUAD cohort, LRP1B mutation showed higher levels of PD-L1 expression among all high-frequency mutations (Fig. 3E). Furthermore, in the analysis of data from LUAD patients who received ICIs in the public database^23^, we found that compared to the WT, patients with LRP1B mutation have higher expression of PD-L1, PD-1, and TMB (Fig. 3F, S2B, S2C), suggesting stronger immune escape capability. Consistently, mutation patients have a shorter overall survival (Fig. 3G). This implies that identifying LRP1B mutation may play a critical part in the pathogenesis of lung cancer and immune response.

### EGFR co-mutation status impacts the TME of LUAD

**Figure 4:**
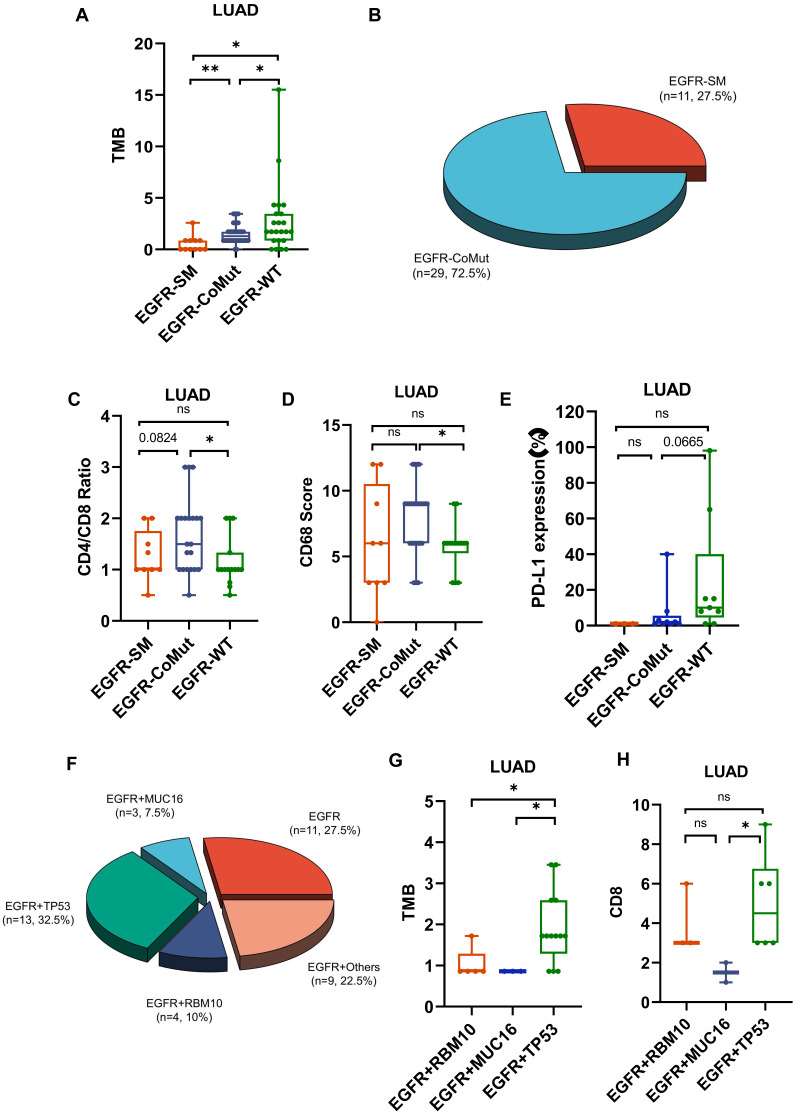
** The impact of EGFR mutation status on the tumor microenvironment (TME).** A. Differences in TMB among EGFR co-mutations (CoMut), single mutations (SM), and wild-type (WT). B. Pie chart shows the distribution of EGFR coMut and SM status. C-E.CD4/CD8 ratio, CD68 scores, PD-L1 expression in EGFR-CoMut, EGFR-SM, EGFR-WT. F. Pie chart depicting the distribution of different types of EGFR co-mutations. G, H. TMB (G), CD8 (H) scores in EGFR+RBM10, EGFR+MUC16, EGFR+TP53 co-occurring mutation statuses.

In high-frequency mutations, TMB and PD-L1 levels were lower in EGFR-mutated patients (Fig. 3C,3E,4A). To conduct a comprehensive investigation, we differentiated two mutation patterns of EGFR, including single mutation and co-mutations. EGFR mutations co-occurring with other genes accounted for 72.5% of all EGFR mutations, while single mutation accounted for 27.5% (Fig. 4B). Further analysis revealed that the co-mutated group had a higher CD4/CD8 ratio (P=0.0195) and CD68 scores(P=0.0101) compared to the EGFR-WT group (Fig. 4C,4D). The downregulation trend of PD-L1 had also been observed in the co-mutated group (P=0.665) (Fig. 4E). However, there was no statistically significant difference between the single EGFR mutation and the EGFR-WT group.

Additionally, we found that the frequently mutated genes TP53, MUC16, and RBM10 often co-occur with EGFR mutations, accounting for 32.5%, 7.5%, and 10% of all EGFR mutations, respectively (Fig. 4F). Among them, EGFR, TP53 co-mutations exhibited significantly higher TMB compared to RBM10 and MUC16, as well as higher CD8 scores (Fig. 4G,4H). These results indicate a lower infiltration of effector T cells and more aggregation TAM in the TME of EGFR co-mutated patients, suggesting an environment more favorable for tumor growth. EGFR and TP53 dual mutation may be the most immunogenic type among all EGFR mutations.

### Distinguishing immune subtypes helps to assess the process of the LUAD

**Figure 5:**
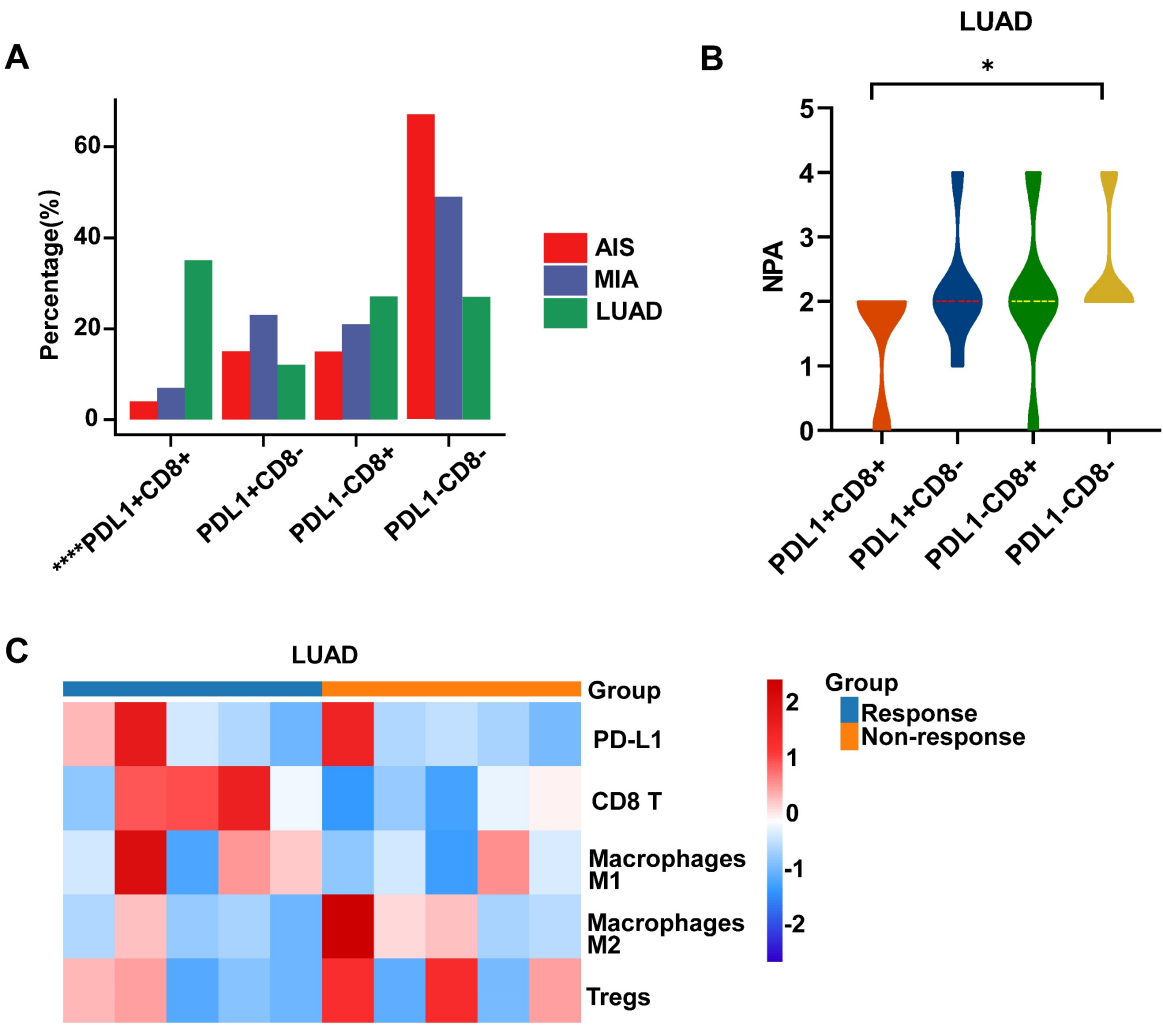
** Analysis of immune subtype features.** A. Proportions of the four immune subtypes in AIS, MIA, and LUAD. B. Distribution comparison of the number of pathway alteration (NPA) among the four immune subtypes in LUAD. C. The immune infiltration in responders and non-responders of LUAD receiving immune checkpoint inhibitors (ICIs).

We further evaluated the immune surveillance status and divided all samples into four immune states based on the expression of CD8 and PD-L1^31^: immune ignorance (PD-L1^-^/CD8^-^), immune tolerance (PD-L1^-^/CD8^+^), intrinsic induction (PD-L1^+^/CD8^-^), and adaptive resistance (PD-L1^+^/CD8^+^). The subtype of PD-L1^+^/CD8^+^ constituted a more significant proportion of tumors in LUAD, in contrast to their occurrence in MIA/AIS (Fig. 5A) (Table S3).

Further analysis was conducted on the distribution of NPA in various immune subtypes. In the PD-L1^-^/CD8^-^ subtype, NPA distribution ranged from 2-4, while in the PD-L1^+^/CD8^+^ subtype, it ranged from 0-2 (Fig. 5B). The specimens in MIA/AIS were not evaluated due to the generally low expression of PD-L1.

By obtaining the transcriptome data of patients who received ICIs from the public database (GSE136961), we divided them into responsive and non-responsive groups and analyzed the PD-L1/CD8 status and immune cell infiltration. Patients with a PD-L1^-^/CD8^-^ status often have poorer efficacy and fewer M1 macrophages, but more M2 macrophages and Treg cells (Fig. 5C). This validates our previous conclusions.

We further divided the patients into the DN group (double-negative: PD-L1^-^CD8^-^) and non-DN group. We analyzed the mutation frequency of high-frequency mutant genes in two groups of LUAD but found no statistical difference (Table S4). These results may indicate that during the progression from AIS to invasive LUAD, although immune infiltration gradually increases initially, as the disease progresses further, the immune escape effect caused by the activation of the PD-L1 pathway is enhanced, ultimately leading to the depletion of immune cells.

### Progression-related mutations promote (PAMs) the development of LUAD by regulating the differentiation of T cells

**Figure 6:**
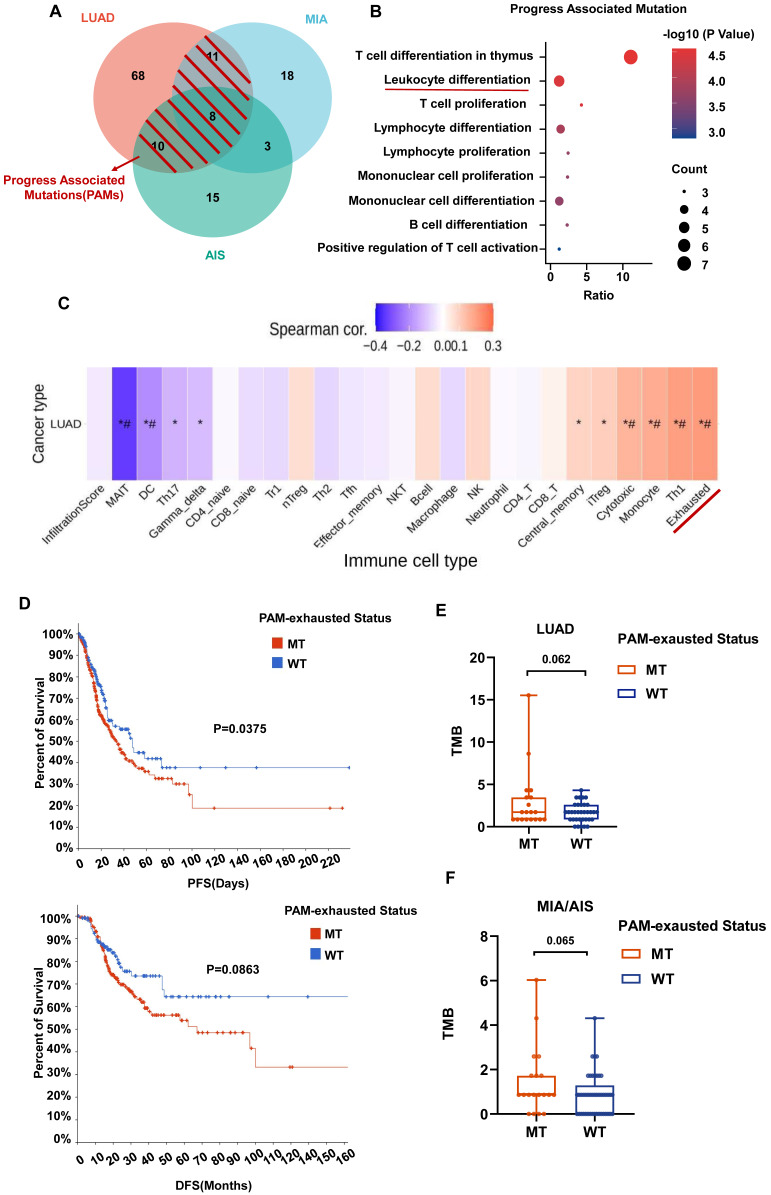
** The role of PAM in the tumor microenvironment (TME) and clinical prognosis.** A. Venn plot showing different and shared genetic mutations in all subtypes of LUAD. The parts highlighted in red represent progress-associated mutations (PAMs). B. Bubble plot of PAMs enrichment analysis in immune-related pathways. B. Correlation heatmap between exhaustion-related mutation (PAM-exhausted) and all immune cell subtypes. PAM-exhausted gene: CARD11, DNMT3A, MUC16, RBM10, TRRAP, ZFHX4. D. Analysis of PAM-exhausted and wild-type (WT) for progression-free survival (PFS)(upper) and disease-free survival (DFS)(under). E, F. TMB levels comparison of PAM-exhausted and WT in LUAD (E) and MIA/AIS (F).

We further defined progression-associated mutations (PAMs) composed of shared mutations in LUAD and MIA/AIS (Fig. 6A). Gene set enrichment analysis was performed using Metascape, and immune-related enriched pathways were selected. It was found that the PAMs were predominantly enriched in pathways associated with Leucocyte differentiation (Fig. 6B). Subsequently, using GSCA, we performed immune genomic analysis and found a strong correlation between a gene set belonging to PAM (CARD11, DNMT3A, MUC16, RBM10, TRRAP, ZFHX4) and exhausted T cells, classifying them as the PAM-exhausted subset (Fig. 6C). We compared the survival outcomes of PAM-exhausted mutant and WT group using Cbiopotal and found that the mutant group exhibited worse PFS and DFS (Fig. 6D).

Finally, in our cohort, although not statistically significant, there was a trend towards a higher TMB in the PAM-exhausted group compared to the WT group, regardless of pathological subtypes (Fig. 6E,6F). These results suggest that in invasive and pre-invasive LUAD, PAMs may contribute to forming a high TMB microenvironment by increasing the presence of exhausted T cells, indicating a poorer short-term clinical prognosis.

## Discussion

The involvement of host immune surveillance has a major significance in the evolutionary process of lung cancer^32^. Throughout the progression of tumor formation, the tumor stroma experiences infiltration by a diverse array of immune cells, with T cells and monocytes/macrophages serving as the primary immune cell types that infiltrate the tumor^33^, ^34^. On the other hand, TMB leads to the generation of new antigens that can be recognized by T cells, resulting in T cell activation and enhanced immunogenicity of the TME^34^. Several studies have utilized TMB and the expression of PD-L1 as prognostic indicators and predictors of the effectiveness of immune checkpoint therapy^35^, ^36^. Our comprehension of pre-invasive and early invasive LUAD remains constrained, particularly regarding the fluctuating distribution of immune cells and the molecular characteristics that may be attributed to these alterations. This study aims to elucidate the evolution of the TME during early-stage LUAD development. The investigation involves examining many factors, including TILs (CD3+, CD4+, CD8+ scores), TAMs (CD68+ scores), TMB levels, and PD-L1 expression. Furthermore, we explore the interplay of these modifications by integrating genomic attributes, including somatic mutations and oncogenetic pathway disruptions.

The findings of our study indicate that the progression of LUAD is associated with a notable elevation in the presence of TILs, TAMs, and TMB. It has been observed that a high TMB may enhance the expression of neoantigens, hence increasing their detectability by the immune system^37^. In this study, TMB was positively correlated with PD-L1 expression across all subtypes, and smokers exhibited a higher TMB level than non-smokers, confirming prior research findings^10^, ^11^. This implies that the immune response has been initiated before the early development of LUAD, and the early surveillance of the immune system attracts innate immune cells, such as macrophages, to the tumor site. Enhanced antigen presentation facilitates the activation of CD8+ T lymphocytes, enabling them to identify and release cytokines, such as IFN-γ, which in turn exert cytotoxic effects on tumor cells, aiming to eradicate them. As mentioned above, the impact exhibits its greatest potency during the process of LUAD invasion^38^.

Furthermore, we demonstrated a noteworthy augmentation in many oncogenic pathways with the progression of the LUAD. Notably, pathways such as PI3K-Akt and Ras exhibited potential associations with the secretion of inflammatory factors^39^, ^40^. This observation suggests that the primary contact between cancer cells and the host's immune system occurs during the initial phases of tumor formation.

The presence of driver mutations with carcinogenic properties is a crucial factor in the beginning of cancer^41^. Recent research findings indicate that these mutations have the potential to impact the immune responses against tumors and modify the progression of tumor development, especially to immunotherapy efficacy^21^, ^42^-^44^. we found that LRP1B had the highest TMB level among all high-frequency mutated genes, which has been reported less frequently before. Considering the potential correlation between TMB and immunogenicity, we speculate that LRP1B could become a new key biomarker for the occurrence of lung cancer and immune therapy efficacy, which is consistent with the results of a previous study^45^.

We then assessed the association between the occurrence of common driver mutations known to cause cancer and the immune milieu within tumors. To begin with, it was observed that tumors harboring EGFR mutations exhibited a comparatively lower TMB compared to groups with other high-frequency mutations and patients with EGFR-WT tumors. This finding implies a reduced level of immunogenicity in LUAD with EGFR mutations. The reported poor therapeutic response observed in patients with EGFR mutations following PD-L1 clinical therapy may be attributed to this phenomenon^46^-^48^. Patients of EGFR mutation patients co-occurring with other genes, particularly TP53, RBM10, and MUC16, exhibited reduced infiltration of effector T cells and a larger population of TAMs compared to EGFR-WT patients. This observation implies a progressive decline in T cell population and an augmentation in the recruitment of TAMs, thereby establishing a beneficial microenvironment for tumor proliferation characterized by inhibitory cytokines and metabolites 49. Our findings provide more evidence to support the existing knowledge that EGFR mutations are linked to a poorer prognosis in immune therapy. Furthermore, our study contributes to a more comprehensive understanding of the subgroups within the EGFR mutation population unlikely to benefit from this therapy. This insight may guide more effective management strategies for these subgroups.

CD8 cell infiltration and PD-L1 expression often affect immune sensitivity and determine whether the tumor is classified as “cold” or “hot”^31^. Our results show that as the pathological progression of LUAD, tumors are more likely to become CD8^+^ /PD-L1^+^This suggests that during LUAD development, there is a gradual upregulation of processes associated with T-cell chemotaxis, immune cell toxicity, and antigen processing. However, this anti-tumor effect may be inhibited by the high expression of PD-L1^31^, ^50^. As the activated tumor pathways increase with LUAD, the tumor microenvironment tends to shift towards a state of immune cell depletion and immune suppression, which will be unfavorable for the prognosis of ICIs. This early identification may enable us to identify the early immune surveillance status of patients and provide assistance in assessing their eligibility for ICIs.

Ultimately, PAMs were identified inside the overlapping region of LUAD and MIA/AIS. By employing gene set enrichment analysis with publicly available datasets, our investigation revealed that PAMs could facilitate the exhaustion of CD8 cells in the microenvironment, which could establish a correlation with an unfavorable short-term prognosis. Numerous factors contribute to the exhaustion of CD8+ T cells, encompassing aberrant tumor neovascularization, dysregulation of adhesion molecules, epigenetic inheritance, and the creation of inhibitory bone marrow cells^51^-^54^. The precise mechanisms underlying this relationship remain uncertain and warrant further intensive exploration.

Although the evolution from AIS to LUAD is assumed to be continuous in the above exploration, they come from different clinical patients, and it is uncertain whether the trajectory of the same patient's development from AIS to LUAD follows what we discussed above. Due to the extremely low recurrence rate of patients with AIS or MIA, it is difficult to obtain all specimens from the same patient, and reacquisition may also be hindered due to the loss of follow-up of patients. For the same reason, we do not have our survival data, and the analysis of its relationship with molecular typing and immune microenvironment can provide significant theoretical support for clinical practice. Thus, multi-center cooperation to obtain larger cohorts and maintain long-term follow-up may be used to solve this problem.

## Conclusions

In summary, we have summarized the changes in TME at the early stage of LUAD and explored the molecular mutation events that may be involved in regulating immune responses. This deepens our understanding of molecular and immune features in developing LUAD. By characterizing CD8 and PD-L1, we can assist in identifying patients with early immune activation or immune evasion and develop corresponding treatment strategies. Furthermore, the discovery of PAMs also aids in predicting the patient's prognosis level and the effectiveness of ICIs, which has significant clinical implications.

## Supplementary Material

Supplementary information, figures and tables.

## Figures and Tables

**Table 1:** Clinicopathological features of enrolled patients

Characteristics	LUAD	MIA	AIS
	N=65	N=52	N=28
Gender			
Male	34(52.3%)	16(30.8%)	9(32.1%)
Female	31(47.7%)	36(69.2%)	19(67.9%)
Age			
<60	39(60.0%)	40(76.9%)	21(75.0%)
≥60	26(40.0%)	12(23.1%)	7(25.0%)
Smoking status			
Yes	17(26.2%)	19(36.5%)	11(39.3%)
No	41(63.1%)	22(42.3%)	16(57.1%)
NA	7(10.8%)	11(21.2%)	1(3.6%)
PD-L1 expression			
High	20(30.8%)	16(30.8%)	5(17.9%)
Low	45(69.2%)	36(69.2%)	23(82.1%)

## Abbreviations

AIS: adenocarcinoma in situ
CIs: confidence intervals
EGFR: epidermal growth factor receptor
GSCA: gene set cancer analysis
IHC: immunohistochemistry
ICIs: immune checkpoint inhibitors
LDL: low-density lipoprotein
LRP1B: low-density lipoprotein receptor-related protein 1B
LUAD: lung adenocarcinoma
MIA: minimally invasive adenocarcinoma
NGS: next-generation sequencing
NPA: number of pathways altered
NSCLC: non-small cell lung cancer
PAMs: progression-associated mutations
RBM10: RNA-binding motif protein 10
PD-1: programmed death-1
PD-L1: programmed death ligand 1
PFS: progression-free survival
PI3K-Akt: phosphatidylinositol 3-kinase-akt
TAMs: tumor-associated macrophages
TILs: tumor-infiltrating lymphocytes
TME: tumor microenvironment
TMB: tumor mutation burden
TPS: tumor proportion score
