# Supplementary material for: An Investigation of the Immune Microenvironment and Genome during Lung Adenocarcinoma Development
Source: J Cancer. 2024 Jan 27;15(6):1687–700. doi: 10.7150/jca.92101 (PMC10869965; doi:10.7150/jca.92101)
Supplement: Supplementary file 1 — Supplementary information, figures and tables. [file jcav15p1687s1.zip › Supplementary Materials/Supplementary Tables and Figures.pdf]

Table S1. Differential immune marker expression in early-stage lung adenocarcinomas based on TMB

Immune marker	Spearman coefficient	P-value
PD-L1	0.17	0.04*
CD3	0.09	0.32
CD4	0.05	0.59
CD8	-0.00232	0.98
CD68	0.19	0.06

*:The difference is significant

Table S2. Distribution of different NPA in LUAD,MIA, and AIS

NPA	LUAD	MIA	AIS	P value
0	7	7	4	0.813
1	4	11	8	0.007*
2	38	30	15	0.994
≥3	18	5	0	< 0.0001*

*:The difference is significant

Table S3. The distribution of each immune subtype in different pathological subtypes

Immune Subtypes	LUAD N=60	MIA N=43	AIS N=27	P-value
PDL1+CD8+ (Immune activation)	21（35.0%）	3（7.0%）	1（3.7%）	P < 0.0001*
PDL1+CD8- (Intrinsic induction)	7（11.7%）	10（23.3%）	4（14.8%）	P > 0.05
PDL1-CD8+ (Immune tolerance)	16（26.7%）	9（20.9%）	4（14.8%）	P > 0.05
PDL1-CD8- (Immune ignorance)	16（26.7%）	21（48.8%）	18（66.7%）	P > 0.05

*:The difference is significant

Table S4. Mutation frequency of high-frequency mutant genes in (double-negative) DN/ non-DN groups of LUAD

Mutant gene	DN group	non-DN group	Number
EGFR	12(40.0%)	18(60.0%)	30
MUC16	1(16.7%)	5(83.3%)	6
RBM10	3(42.9%)	4(57.1%)	7
TP53	2(33.3%)	4(66.7%)	6
ERBB2	1(50%)	1(50%)	2

DN group: PD-L1$^-$CD8$^-$ subtype

non-DN group: PD-L1$^+$CD8$^+$, PD-L1$^+$CD8$^-$, PD-L1$^-$CD8$^+$ subtypes

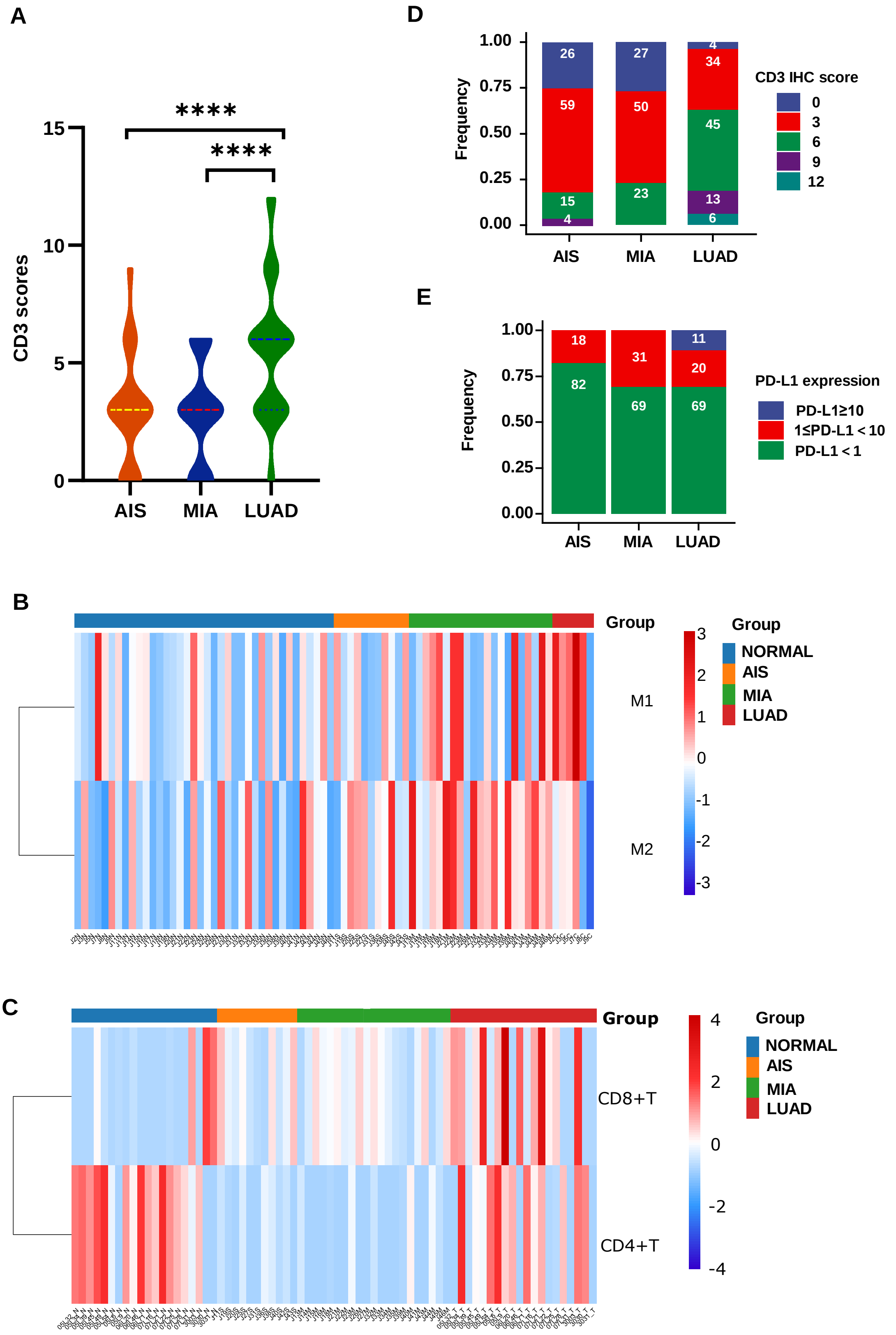
A
****
****
CD3 scores
15
10
5
0
AIS
MIA
LUAD
D
Frequency
1.00
0.75
0.50
0.25
0.00
26
59
15
4
27
50
23
4
34
45
13
6
AIS
MIA
LUAD
CD3 IHC score
0
3
6
9
12
E
Frequency
1.00
0.75
0.50
0.25
0.00
18
82
31
69
11
20
69
AIS
MIA
LUAD
PD-L1 expression
PD-L1≥10
1≤PD-L1 < 10
PD-L1 < 1
B
Group
M1
M2
3
2
1
0
-1
-2
-3
Group
NORMAL
AIS
MIA
LUAD
C
Group
CD8+T
CD4+T
4
2
0
-2
-4
Group
NORMAL
AIS
MIA
LUAD

Figure S1. Immune characteristics in early-LUAD

A. Comparison of CD3+ score in AIS, MIA, and LUAD. B, C. Macrophages(B) and T lymphocytes(C) in AIS, MIA, LUAD, and adjacent normal tissue. D. The distribution of different CD3 scores in all pathological types. E. The distribution of different level of PD-L1 expression in all pathological types.

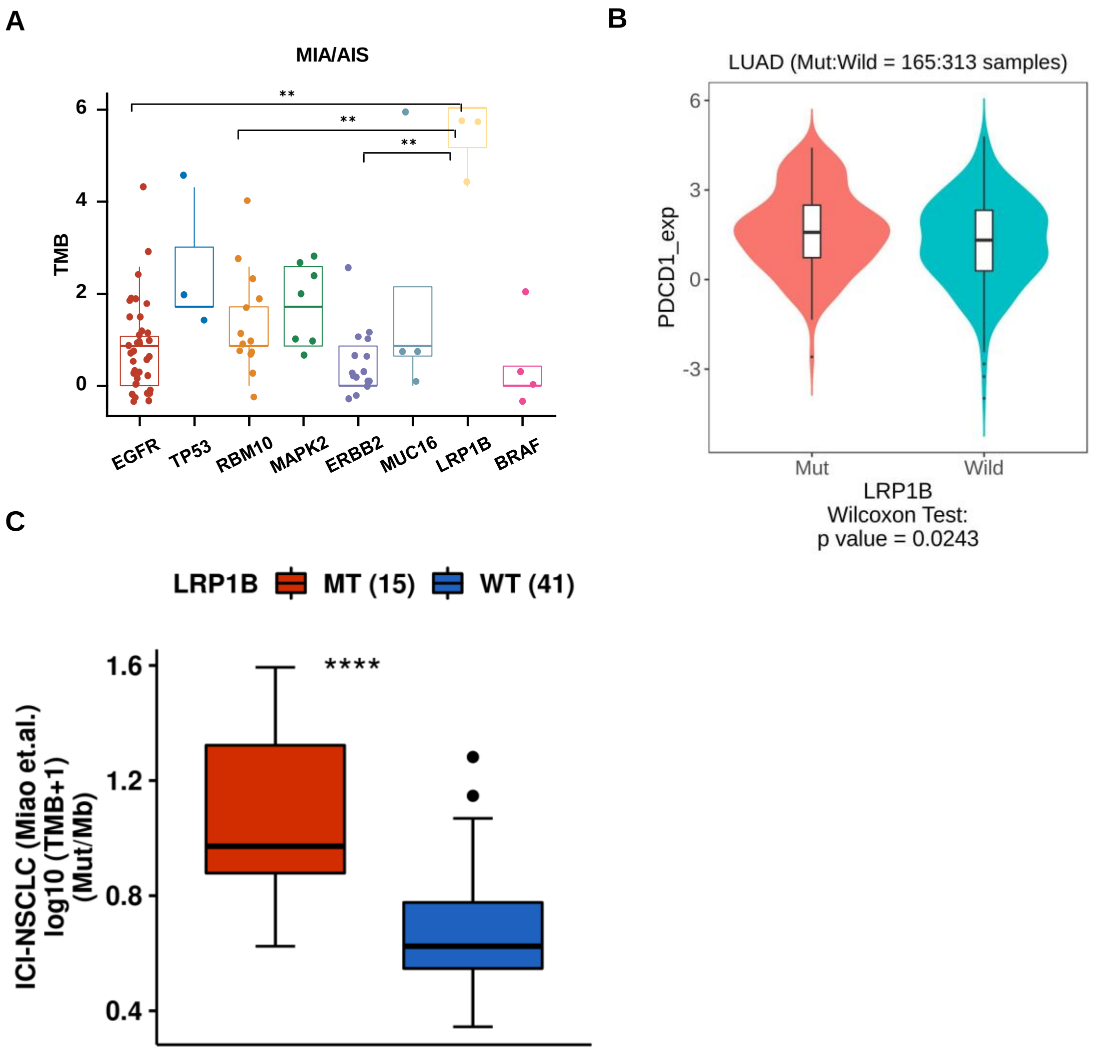
A
MIA/AIS
TMB
6
4
2
0
**
**
**
EGFR
TP53
RBM10
MAPK2
ERBB2
MUC16
LRP1B
BRAF
B
LUAD (Mut:Wild = 165:313 samples)
PDCD1_exp
6
3
0
-3
Mut
Wild
LRP1B
Wilcoxon Test:
p value = 0.0243
C
LRP1B
MT (15)
WT (41)
ICI-NSCLC (Miao et.al.)
log10 (TMB+1)
(Mut/Mb)
1.6
1.2
0.8
0.4
****

Figure S2. Genomic characteristics in early-LUAD

A. TMB levels of samples with high-frequency mutations in MIA/AIS. B. The PD-1 expression in LRP1B-mutated and wild-type LUAD patients. C. The TMB level of LRP1B-mutated and wild-type LUAD patients receiving immune checkpoint inhibitors(ICIs).
